# Occurrence and size distribution of silver nanoparticles in wastewater effluents from various treatment processes in Canada

**DOI:** 10.1007/s11356-021-15486-x

**Published:** 2021-07-29

**Authors:** Christian Gagnon, Patrice Turcotte, François Gagné, Shirley Anne Smyth

**Affiliations:** Science & Technology Branch, Environment and Climate Change Canada, 7th Floor, St. Lawrence Centre, 105 McGill Street, Montreal, QC H2Y 2E7 Canada

**Keywords:** Ag, Silver nanoparticles, Single particle ICP-MS detection, Wastewater

## Abstract

The occurrence of silver (Ag) in urban effluents is partly associated with the increasing use of silver nanoparticles (Ag NPs) as an antiseptic agent in various consumer products. Distinction among Ag forms must be taken into account in the assessment of exposure and toxicological effects to aquatic organisms. Wastewater treatment processes effectively remove Ag particles and colloids (mostly > 95%), but this still leaves notable concentrations (in order of ng/L) escaping to effluent-receiving waters. Total suspended Ag concentrations in various studied effluents ranged from 0.1 to 6 ng/L. The purpose of this study was then to measure and characterize Ag NPs in urban effluents for their concentrations and size distribution using the single particle ICP-MS technique (SP-ICP-MS). Wastewater influents and effluents from various treatment plants—from aerated lagoons to advanced treatment technology—were collected for three sampling days. Our results showed the presence of Ag NP in all samples with concentrations reaching 0.5 ng/L on a mass basis. However, on a particle number basis, Ag NP concentrations (expressed in particle/mL) in the 20–34-nm fraction (up to 3400 particles/mL) were much more abundant (> 700%) than in the > 35-nm larger fraction. The proportion of Ag at the nanoscale (1–100 nm) represents less than 8% of the total suspended Ag for all effluent samples, regardless of their origins. A significant correlation (linear regression: *r*^2^ > 0.7) was observed between Ag NP and total suspended Ag concentrations in investigated effluents. Because Ag nanotoxicity is size dependent, the determination of size distribution and exposure concentration on a particle number basis is urgently needed for risk assessment of this class of nanoparticles.

## Introduction

The presence of silver in urban effluents is associated with the increasing use of silver nanoparticles (Ag NPs) as an antiseptic agent (Musee et al. 2011; Dale et al. 2015). Indeed, Ag NPs are embedded in clothing, food containers, medical materials, and others (Bhatt and Tripathi 2011). The majority of Ag NPs in consumer products go down the drain and reach wastewater treatment plants (Kaegi et al. 2011). Although most wastewater treatment processes effectively (95%) remove both particulate and dissolved Ag, they still release notable concentrations (in order of few ng/L) to effluent-receiving waters (Li et al. 2013).

The toxicity of Ag NPs on aquatic organisms could change markedly upon passing through municipal wastewater treatment plants (WWTPs), as their specific properties can be modified by physical and chemical transformations in WWTPs (Kaegi et al. 2011; Bruneau et al. 2016). Alterations of Ag NPs can be caused by a number of possible transformation processes such oxidation and sulphidation (Kaegi et al. 2011; Kraas et al. 2017; Lowry et al. 2012; Georgantzopoulou et al. 2018). When treated wastewater effluents are released in streams, they likely still contain Ag in dissolved, particulate, and NP forms. Dissolved ionic silver (Ag+) and small complexes are considered highly available Ag forms for aquatic organisms (Khan et al. 2012; Gagne et al. 2013).

From the environmental risk management perspective, it is important to better understand the size distribution of released Ag in nanoparticle forms since they could become more toxic than the bulk ion counterpart (i.e., Ag(I) because of the high surface area and reactivity (Bhatt and Tripathi 2011). Key physicochemical properties such as the particle size have been associated with the toxicity of engineered NPs (Jiang et al. 2009) where smaller nanoparticles tend to penetrate more quickly into cells, reaching the nucleus in some cases and producing more toxicity owing to increased surface-area-to-volume ratio. Cytotoxicity studies investigating the size-dependent cellular toxicity of Ag NPs also reported particle size–dependent cytotoxic effects and suggested careful consideration of particle size, not only concentrations, in testing design (Kim et al. 2012). Ag NPs have the potential to cause ecotoxicological effects due to both being within the nano size range and also from dissolution to Ag+ states (Jiang et al. 2009; Cunningham et al. 2013).

Their environmental monitoring, both in effluents and in their receiving aquatic ecosystems, requires a sensitive analytical approach that will allow the differentiation of the Ag NP from the dissolved Ag. Mass spectrometry coupled with argon plasma (ICP-MS) is commonly used for the analysis of total Ag at low levels (MDL 0.2 ng/L). Recent technological advances of these instruments can also be used to distinguish between the nanoparticulate and dissolved Ag phases using the single particle detection approach (SP-ICP-MS). This technique involves collecting a series of measurements each millisecond for at least 1 min where dissolved and abundant elements evenly distributed in the solution generates a constant signal, while the passage of Ag NP in plasma produces short bursts (0.5 ms) of intense signal. The intensity of this signal is proportional to the size of the particle (Mitrano et al. 2012; Pace et al. 2011; Tuoriniemi et al. 2012). While such analytical approach is key for Ag speciation in surface waters, investigating Ag NP in complex wastewater samples remains a challenge. For a more accurate detection of Ag NP by SP-ICP-MS, the technique was modified by the use of 109 Ag isotope detection avoiding zirconium oxides—generated by Zr particles—isobaric interferences in ICP plasma (Turcotte and Gagnon 2020).

A few studies have investigated the release of silver from municipal WWTPs based on particulate size distribution (e.g., Johnson et al. 2014; Li et al. 2016). Nanoscale Ag particles (defined as n-Ag-Ps) were specifically analysed by different size-based techniques, such as cloud point extraction (CPE), ion-exchange resin (IER), or ultrafiltration (UF) (Li et al. 2013; Gagnon 2018). The colloidal forms of n-Ag-Ps include forms such as Ag nanoparticles and Ag_2_S. While significant concentrations of detected n-Ag-Ps can be seen as a sign of Ag NP occurrence in wastewaters (Li et al. 2013; Johnson et al. 2014), more investigations of the Ag colloidal fraction are needed with better discrimination in the identification of Ag NP.

This is the first study on the characterization of NP Ag releases from different WWTPs using single particle–mode ICP-MS, a technique adapted for the specific detection (Ag atoms based) of NPs (Turcotte and Gagnon 2020; Mitrano et al. 2012). The technique allows distinguishing Ag forms where each Ag particle, containing high Ag atom density, produces an intense peak, compared to other forms, in mass spectrometry. Various municipal WWTPs, using mechanical or biological treatments (activated sludge, nutrient removal) were sampled with the aim to determine their Ag removal and releases of different Ag forms, including particulate, colloidal, and nanoparticulate forms.

The objective of this investigation was therefore to examine the occurrence and the size distribution of Ag released from municipal effluents. Municipal effluents from ten municipalities using different treatment processes—from aerated lagoons to advanced biological treatments—were sampled across Canada. Moreover, untreated wastewaters were also collected to determine the overall Ag removal at WWTPs.

## Methods

### Wastewater sampling collection

Samples of wastewater raw influent and final effluent were collected from 10 WWTPs of different treatment technologies across Canada in 2017, targeting dry weather conditions periods as possible. Table 1 summarizes the various types of treatment of these WWTPs that included aerated lagoons (AL), secondary biological treatments using conventional activated sludge (ST), and two advanced biological nutrient removal treatments with tertiary filtration (AT). Both influent and effluent samples were collected for three consecutive days (*n* = 3) using Hach Sigma 900 refrigerated autosamplers (Hach Company, Loveland CO, USA) to obtain 24-h equal volume composite samples at 400 mL every 15 min and better consider effluent fluctuations. Wastewater samples were subsampled in 1-L high-density polyethylene bottles and shipped on ice to the laboratory. Subsamples of influent and effluent were transferred to 50-mL polypropylene tube, and frozen at − 20 °C, without acid preservative solution, until the day of analysis. Samples were slowly thawed in a cold-water bath, in order to avoid over-heating of the samples, and then placed in an ultrasonic water bath for 5 min at 20 °C. For total Ag determination in influents and effluents, samples were acidified with 1% HNO3 and 1% H2O2, in order to evaluate removal efficiency of the various wastewater treatment plants. Total suspended solids (TSS) were analysed by the Environment and Climate Change Canada’s National Laboratory for Environmental Testing according to standard methods (APHA 2005) and removal efficiency for those quality-control parameters are summarized in Table 1.

**Table 1:** Total Ag concentrations (ug/L) in wastewater influents and effluents from various treatment plants (WWTP) and removal efficiency. TSS: total suspended solids. *: values in parentheses are concentration variability for three sampling days

WWTP type	Plant ID	Flow m3/day	Filtration	% TSS removal	Influent Total Ag		Effluent Total Ag		% Ag removal
Aerated lagoon	TB	25,760	None	96	0.147	(0.013)*	0.025	(0.003)*	83
	JL	27,650	None	92	0.074	(0.025)	0.005	(0.001)	93
Secondary *(Activated sludge)*	SB	1040	Sand	99	0.202	(0.099)	0.002	(0.003)	99
	WL	37,890	None	98	0.254	(0.150)	0.003	(0.001)	99
	PD	38,750	None	99	0.214	(0.190)	0.002	(0.000)	99
	HG	2170	Sand	99	0.151	(0.099)	0.005	(0.002)	97
	PA	3990	None	98	0.129	(0.045)	0.003	(0.002)	98
	PW	35,100	None	99	0.292	(0.043)	0.003	(0.001)	99
Advanced with *filtration (biological nutrient removal)*	E	35,770	Cloth disc	99	0.235	(0.010)	0.009	(0.001)	96
	PN	11,720	Cloth disc	99	0.402	(0.423)	0.008	(0.000)	98

### Total Ag analysis in wastewaters

Mass spectrometry coupled with argon plasma (ICP-MS) was used for the analysis of total silver at low levels (LOD, 0.2 ng/L). To assess Ag total concentration in solution, the instrument was calibrated with ion standards (SCP Science). The Ag ionic standard was purchased from SCP Science (Baie-d’Urfée, Montréal, Canada). By serial dilutions, solutions of 0.1, 0.5, and 1.0 μg/L were prepared in 1 % HNO3 (Baseline grade, SeaStar, Vancouver, Canada). The precision, expressed as coefficient of variation was 12% or better and detection accuracy, expressed as % of recovery, was between 82 and 100%.

### NP Ag analysis by SP-ICP-MS

**Figure 1:**
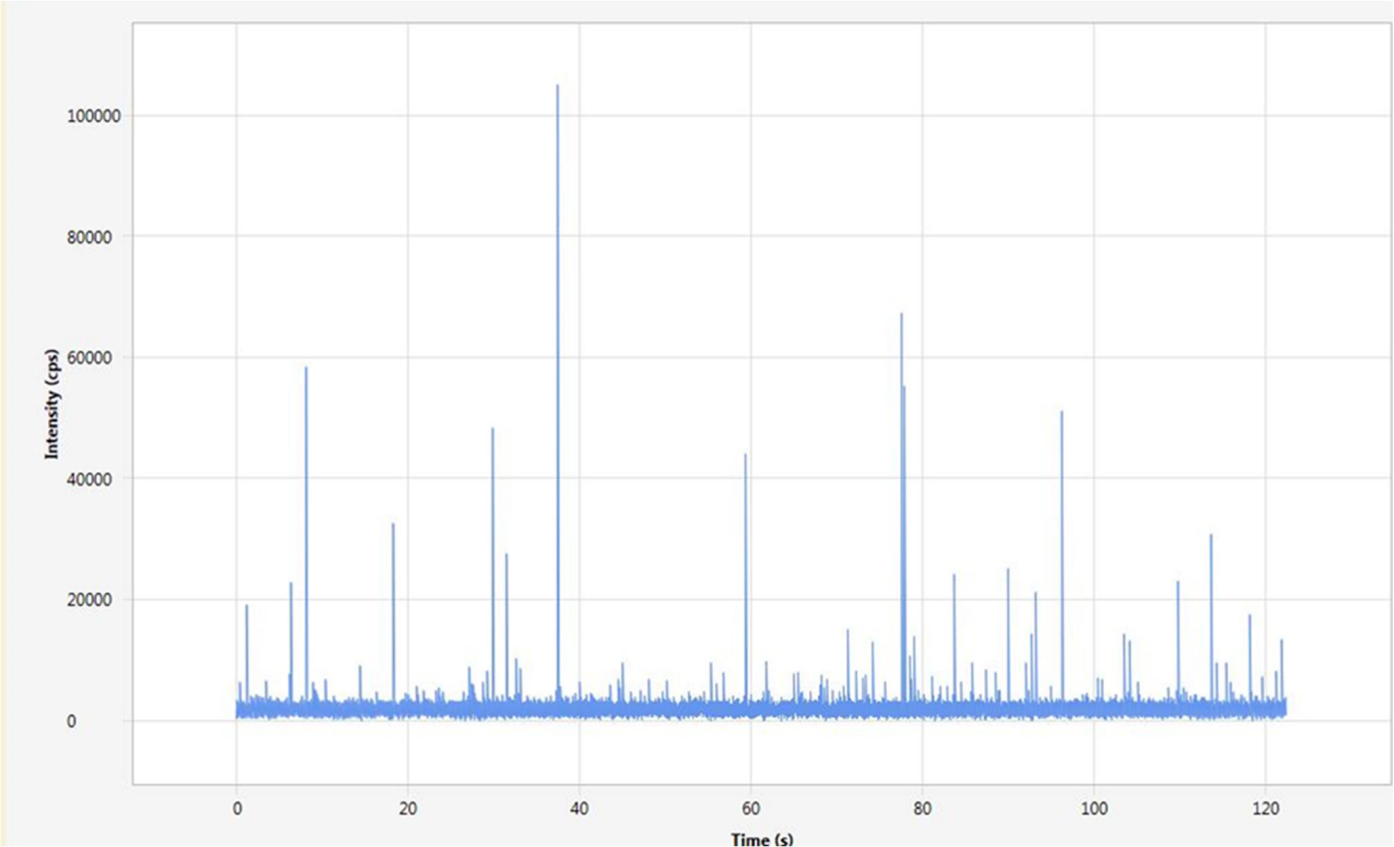
Example of analytical detection of silver (Ag NP and dissolved Ag) by single particle ICP-MS in municipal wastewater effluent samples

Ag NPs in urban effluents were evaluated by ICP-MS using the single particle technique (SP-ICP-MS) with a Thermo™ Model ICAP-RQ. Prior to analysis, the samples were transferred to 14-mL polypropylene tubes and not filtered as we chose not remove particles in solution that could contain Ag NP, but allowed partial sedimentation for 1 h of aggregates and large particles. With this procedure, the variation coefficient for total suspended Ag was better than 5% between sample duplicates. Because the main goal of this study was to evaluate the discharge of Ag NP, the Ag fraction evaluated was what remained in suspension. Thus, Ag nanoparticulate and dissolved phases were characterized on suspension samples using the single particle detection approach (SP-ICP-MS). The analysis of Ag NP by the SP-ICP-MS technique makes it possible to measure the number and size of particles. The passage of Ag NP in plasma is characterized by an intense signal (peak) for a very short period of time (0.5 ms) while the signal of dissolved Ag(I) is evenly distributed as constant signal (Fig.1). The number of events is directly correlated with the number of nanoparticles in the solution and the pulse intensity is a function of the particle size which is related to the number of targeted isotopes (Degueldre et al. 2004; Mitrano et al. 2012; Pace et al. 2011; Tuoriniemi et al. 2012). For a more accurate detection of Ag NP in urban effluents by SP-ICP-MS, the technique was modified by the use of 109 Ag isotope detection to avoid zirconium particle/colloid interferences. Zirconium is known to form oxides that interfere with the silver detection with 107 isotopes and to a lesser extent with 109. When Zr particles are present, the oxide interference signal is like Ag NP pulses (Turcotte and Gagnon 2020). The urban effluents contain zirconium particles and those induce false positives for small Ag NPs. We have defined a size threshold that included false positives probability of 20 nm. Thus, particles with size below 20 nm were not considered as Ag NPs. The data processing was done with a Thermo™ NP-Quant software according to the equations defined by Pace et al. (2011). The acquisition time was 120 s and the dual time 2.5 ms allowing the integration of the entire signal peaks. The intensity for 1 μg Ag/L was 185000 cps. The transport rate, as defined by Pace et al. (2011) and Mitrano et al. (2012), which relates the measured concentration (particle/mL) of Ag NP in the instrument to that of the sample, was approximately 7.5%. This was evaluated with certified 80nm Ag NP and 80nm Au NP purchased from TED PELLA ™. The standards of 4.2 ng/L of NP-Ag-80 and NP-Au-80 were prepared in a solution (Milli-Q water) of tri-sodium citrate (2 mM). The SP-ICP-MS technique also makes it possible to quantify the total concentration of silver in solution, the dissolved phase (ionic and small complexes), and the size and number of Ag NP. The detection limit for dissolved silver was 0.1 ng/L. The minimum number of Ag NP that can be detected was 15 particles/mL of sample. This same minimum number of detection, expressed in ng of Ag NP/L, when applied to only particles of 20 nm, was equivalent to 0.0003 ng/L. For the fraction of Ag NP 20–34 nm expressed in ng/L, RDS was better than 15% while expressed in part/mL it was 18%. For the fraction > 35 nm, they were better than 49 and 44%, respectively. The total suspended Ag concentration, Ag particle number, and ng/L concentration were all measured during the same analysis.

We operationally ranked our results in three classes: total suspended silver (without aggregates and large particles), NP > 35 nm, and small Ag NP = 20–34 nm. The summation of the two fractions of Ag NP in ng/L divided by total suspended silver allows estimating the proportion (%) in mass of Ag NP in the sample analysed by SP-ICP-MS.

## Results and discussion

### Silver removal at WWTPs

Concentrations of total Ag ranged from 0.15 to 0.40 μg /L in influent samples (before treatment) and from 0.003 to 0.025 μg/L in the treated effluent samples (Table 1). The highest total Ag concentrations (0.025 μg/L) reported for the studied effluents were observed in an aerated lagoon. Total Ag concentrations (Ag NP and Ag(I)) were previously measured in effluent samples from a WWTP in California, USA, with high values ranging from 0.04 to 0.07 μg/L (Cervantes-Avilés et al. 2019). The authors reported removal of 76 and 96% of the colloidal Ag fraction by secondary and tertiary ultrafiltration treatment processes, respectively. In this study, the removal efficiencies were all above 80%, often reaching > 96% (Table 1). As a result, concentrations of total Ag in treated wastewater effluents were relatively low with values lower than 25 ng/L [0.025 μg/L]. Reported mean values for the removal of particulate Ag at various British WWTPs were around 98%, and to a lesser extent, near 50% of removal for the colloidal forms, being defined here by ultra-filtration with 2-nm cut-off (Johnson et al. 2014). The silver particulate form is most effectively removed with the sludge settlement process. Such results indicate potential escape of certain nanosilver fractions from WWTPs (Johnson et al. 2014). Total Ag removal performance among the different types of WWTPs investigated (aerated lagoon, activated sludge with or without filtration, biological nutrient removal with filtration) were all high (< 80%) when compared to the corresponding non-treated raw wastewaters. Hence, total Ag removal is treatment invariant with little exception for aerated lagoons. Trends in Ag removal were similar to those of total suspended solids (TSS) removal with the lowest values also observed for aerated lagoons (Table 1).

### Release of Ag nanoforms from WWTP effluents

**Figure 2:**
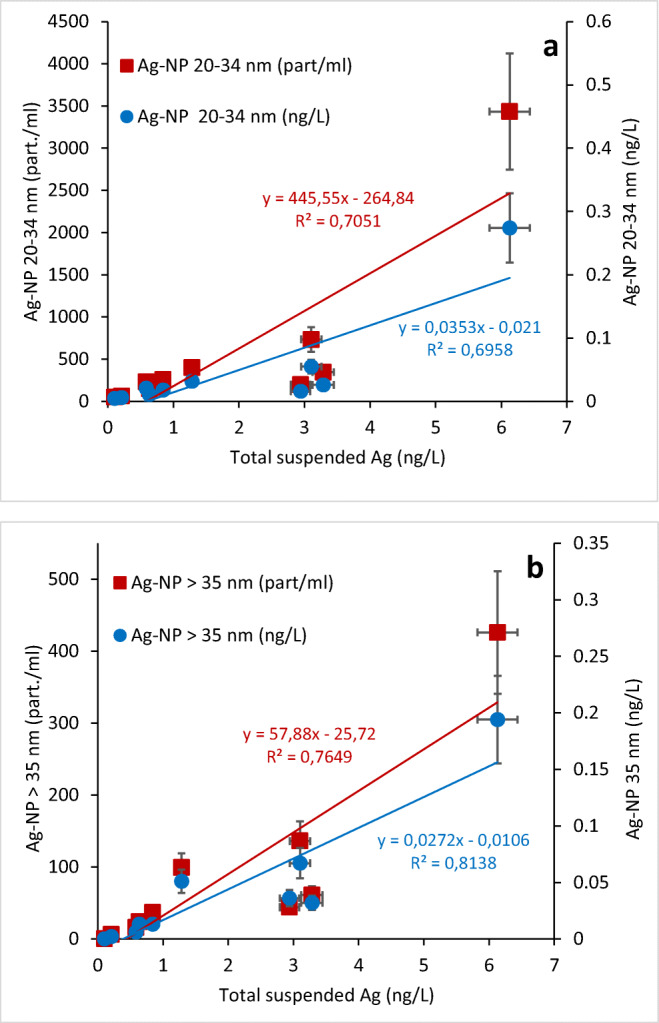
Distribution of Ag NP size fractions (20–34 and > 35 nm) in wastewater. **a**). Ag Np 20–34-nm fraction; **b** Ag NP > 35-nm fraction

In the process of determining the presence of Ag at the nanoscale (including all colloidal forms), total suspended Ag was first measured by ICP-MS in effluent samples with concentrations ranging from 0.1 to 6.0 ng/L (Table 1). These first values agree well with the German study’s mean value of 5 ng/L for nanoscale and colloidal Ag estimated by an extraction based on cloud point technique (Li et al. 2013). Based on the single particle ICP-MS technique, the presence of Ag NP was observed in all effluent samples. Results were arbitrarily operationally ranked in three classes: total suspended silver, Ag NP 20–34 nm, and Ag NP > 35 nm expressed as particle number (part./mL) or concentration (ng/L) (Fig 2). When expressed in particle/mL concentration, Ag NP in the 20–34-nm fractions were much more abundant than in the > 35-nm larger fraction. On the other hand, when concentrations of Ag NP are expressed in ng/L, this trend was not highly observed as particles in the 20–34-nm fraction are lighter (Fig. 2). Due to different particle mass, a 40-nm spherical particle is 4 times heavier than a 25-nm particle. The summation of the two fractions of Ag NP in ng/L divided by total suspended silver allows estimation of the percentage in mass of Ag NP in the sample (Table 2, Fig. 2). Ag NP measured by SP-ICP-MS ranged from 0.02 to 0.47 ng/L representing 1.7–7.6 % of total suspended Ag in effluents. In a study on British wastewater treatment plants, the mean concentration of colloidal (2–450 nm) silver, which includes nanosilver, was reported to be 12 ng/L in influents and 6 ng/L in effluents (Johnson et al. 2014). For comparison in the same samples, particulate silver (> 450 nm) was rather within the μg/L concentration range with mean values of 3.3 and 0.08 μg/L for influents and effluents, respectively. The contribution of Ag NP would therefore represent less than 8% of the suspended Ag released in effluents (Table 2). The proportion of silver in “nanometric forms” expressed in mass was, on average, 4% for all effluent samples, regardless of the type of wastewater. The concentration of Ag NP expressed in mass increased in relation (*R*^2^ = 0.96) with total Ag concentration in effluents. The highest NP proportion values were observed in the aerated lagoon effluents (Table 2). These results may suggest that treatments by lagoons are less effective in removing Ag NP from effluents. Hence, the size distribution of Ag could be influenced by treatment strategies, where aeration lagoons discharge higher proportion of Ag NP. In the same way, lower removal of total Ag was also observed for those treatments by lagoons (Table 1). Relationships (linear regression: *R*^2^ > 0.7) were observed, at some extent, between Ag NP and total suspended Ag concentrations in wastewater effluents (Fig. 2). Similar trends were also noted when the concentration of Ag NP is expressed as particle number/mL (Fig. 2). Implicitly, concentrations of both, small and larger size particle fractions, were directly proportional to total suspended Ag concentrations. All these trends seem indicate that Ag in effluents may have a common origin.

**Table 2:** Mean Ag NP concentrations in wastewater effluents from various wastewater treatment plant types (WWTPs)

WWTP type	Plant ID	Total suspended Ag (ng/L)	∑ Ag NP (ng/L)	% as NP
Aerated lagoon	TB	6.129	0.468	7.6
	JL	1.284	0.084	6.6
Secondary	SB	0.104	0.005	4.8
	WL	0.585	0.027	4.6
	PD	0.208	0.007	3.4
	HG	2.940	0.052	1.8
	PA	0.635	0.023	3.6
	PW	0.842	0.031	3.7
Advanced with filtration	E	3.284	0.057	1.7
	PN	3.101	0.122	3.9

Biological and physical processes in the secondary treatment generally removed more than 75% of the colloidal Ag fraction (Cervantes-Avilés et al. 2019). A study using cloud point extraction (CPE) method as the detection technique reported that nanoscale Ag particles (n-Ag-Ps) concentrations, which included all colloidal forms, in the influent were as high as < 1500 ng/L and decreased (∼ 35%) in concentrations after primary treatment (physical clarification), indicating that initial treatment steps contributes to the n-Ag-Ps removal (Li et al. 2013). Afterward, further biological treatment (activated sludge) effectively removed those colloidal forms, which resulted in low concentration (0.7–11.1 ng/L) of n-Ag-Ps in the effluents. Using the CPE technique, Li et al. (2016) demonstrated that more than 96% of nanoscale Ag particles (Ag-b-NPs) from wastewater influent are removed through WWTPs. In a laboratory experiment with municipal wastewater, spiked Ag NP was mostly observed as transformed sulphidized forms (Kim et al. 2010). Nanoscale Ag_2_S particles were identified in sewage sludge using high-resolution transmission electron microscopy. Based on transformation kinetics, sulphidation of Ag NP would likely occur at NP surface as a result of transformed Ag NP (He et al. 2019) which are also detected by SP-ICP-MS. The ultimate fate of most Ag material (up to 90%) would be accumulated at the end of wastewater treatments in sludge as sulphides (Kaegi et al. 2011; Kim et al. 2010; Tiede et al. 2010; Thalmann et al. 2014).

Despite low concentrations of Ag escaping as NPs, such additional Ag sources—even in ng/L concentration ranges—from uses of nanotechnology and ultimately released in municipal wastewaters can be considered as cumulative contamination sources. These relatively low Ag concentrations are continually discharged at variable loads according to the size of the city and effluent flow. Even after high particulate and colloidal Ag removal efficiency (> 95%) by treatment plants, effluents were reported to release much lower concentrations of nanoscale Ag (up to 12 ng/L), but still a relative Ag contribution to effluent-receiving surface waters (Li et al. 2013; Johnson et al. 2014). As a result, colloidal Ag forms were tracked down along a major effluent dispersion plume (Gagnon et al. 2006), confirming the need for further physicochemical characterization. Because of the continuous release of Ag by municipal effluents, this represents an Ag source to aquatic environments where characterization of the size distribution of released Ag forms (Brar et al. 2010; Kaegi et al. 2013; Government of Canada 2016) down to the nanoscale is therefore needed for risk assessment.

A laboratory study carried out with river waters showed that Ag NP slowly degrades (half-life: 12 days) by releasing a little dissolved Ag(I) at a time (Gagnon 2018). Those experiments on transformation kinetics concluded that Ag NP degraded into smaller NP and steadily released over time Ag+ ion and small complexes as final degradation products under natural water conditions. Thus, measured Ag forms remaining in treated wastewater are likely the result, to some extent, of degradation processes and contribute to the overall Ag contamination of effluent-receiving waters. Following exposure to test media containing Ag NPs, silver ions (Ag(I)) released from Ag NPs were typically targeted to be the major pathway leading to body burden (Kühr et al. 2018). No Ag ions, however, were significantly detected (< 0.1 ng/L) in the dissolved phase of such complex matrices with abundant organic and inorganic Ag ligands. Nevertheless, all Ag NP forms and their transformation products—including complexed and adsorbed ion forms—contribute to water contamination through the use of silver-containing consumer products. Different studies have shown that ingested particulate Ag forms may also be bioavailable for aquatic organisms (Croteau et al. 2011; Gomes et al. 2013; Bruneau et al. 2016). For instance, with mussels, gills are the major organs for the uptake of dissolved Ag, whereas Ag NP aggregates are primarily taken up by the gut or digestive gland. This points out the need to consider all NP aggregation and dissociation transformations that could influence exposure pathways and potential bioavailability.

**Figure 3:**
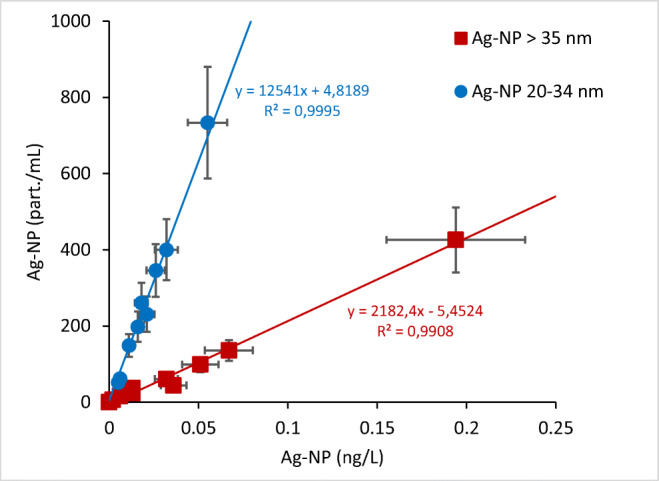
Comparison between Ag NP concentrations expressed as mass (ng/L) and particle numbers (part/mL)

While the observed concentrations in mass (ng/L) were within similar value ranges for small and large size fractions, more different values (> 700%) were obtained between the two fractions in terms of quantity of NP (particles /mL) (Fig. 3). Up to 3000 particles/mL were measured in the small size fraction 20–34 nm, while no more than 450 particles /mL in the large NP fraction (> 35 nm). As a general pattern in term of NP size distribution, an average of 730% enrichment of the small NP fraction was estimated for the investigated wastewater samples. Such information on the relative occurrence of the smallest NP is key for assessing the real impacts of discharged Ag NP. Several studies have shown size-dependent toxic effects. For example, Contreras et al. (2014) found that the small Ag particles (2 nm) had a notable toxicological effect on nematode while larger particles had different, lower-extent effects. Therefore these studies highlighted the need for size characterization, not just the exposure concentrations, for a better exposure and risk assessment of Ag NP. In standard assessment method development (OECD 2020), such size-based information like particle number concentration (in contrast to mass concentration) is recommended for other exposure metrics that can be used in risk assessments and better understanding the mode of action of different NP forms, i.e., that size, total Ag concentration, and particle density should be included in risk assessment schemes of inorganic NPs.

## Data Availability

N/A. Data supporting the results reported in the article can be found in the figures and tables included in this paper.
